# Occult hypoperfusion and changes of systemic lipid levels after severe trauma: an analysis in a standardized porcine polytrauma model

**DOI:** 10.1007/s00068-022-02039-1

**Published:** 2022-07-12

**Authors:** Yohei Kumabe, Yannik Kalbas, Sascha Halvachizadeh, Michel Teuben, Nikola Cesarovic, Miriam Weisskopf, Andreas Hülsmeier, Thorsten Hornemann, Paolo Cinelli, Hans-Christoph Pape, Roman Pfeifer

**Affiliations:** 1 Department of Trauma, Institute for Clinical Chemistry, Zurich University Hospital, Zurich, Switzerland; 2 Department of Surgical Research, Harald Tscherne Laboratory for Orthopaedic and Trauma Research, Zurich University Hospital, Zurich, Switzerland

**Keywords:** Lipidomic analysis, Lipid, Polytrauma, Haemorrhagic shock, Occult hypoperfusion, Resuscitation

## Abstract

**Background:**

Occult hypoperfusion describes the absence of sufficient microcirculation despite normal vital signs. It is known to be associated with prolonged elevation of serum lactate and later complications in severely injured patients. We hypothesized that changes in circulating lipids are related to responsiveness to resuscitation. The purpose of this study is investigating the relation between responsiveness to resuscitation and lipidomic course after poly trauma.

**Methods:**

Twenty-five male pigs were exposed a combined injury of blunt chest trauma, liver laceration, controlled haemorrhagic shock, and femoral shaft fracture. After 1 h, animals received resuscitation and fracture stabilization. Venous blood was taken regularly and 233 specific lipids were analysed. Animals were divided into two groups based on serum lactate level at the end point as an indicator of responsiveness to resuscitation (<2 mmol/L: responder group (R group), ≧2 mmol/L: occult hypoperfusion group (OH group)).

**Results:**

Eighteen animals met criteria for the R group, four animals for the OH group, and three animals died. Acylcarnitines showed a significant increase at 1 h compared to baseline in both groups. Six lipid subgroups showed a significant increase only in R group at 2 h. There was no significant change at other time points.

**Conclusions:**

Six lipid groups increased significantly only in the R group at 2 h, which may support the idea that they could serve as potential biomarkers to help us to detect the presence of occult hypoperfusion and insufficient resuscitation. We feel that further study is required to confirm the role and mechanism of lipid changes after trauma.

## Introduction

Complications after severe trauma have been associated with an imbalanced systemic reaction to the multiple insults occurring after trauma [1]. The typical pathophysiological course of traumatized patients undergoing ICU treatment is characterized by a systemic inflammatory response that involves complex interactions across the haematologic, inflammatory, endocrine, and neurological systems, thus aggravating initial damage caused by hypoperfusion and reperfusion [2–4]. Despite multiple efforts to understand the underlying pathophysiology of postinjury complications, full understanding of the mechanisms has not yet been achieved [2–8]. Observational studies indicate that occult hypoperfusion might be present, even in stable macrocirculation [9–15]. Moreover, others have described that not all patients directly respond to resuscitation strategies and may be at risk for later complications [16–18].

In addition, post-traumatic fat embolism is an ongoing clinical problem in patients with acute trauma and long bone fractures [19]. It is mainly caused by intravasation of bone marrow fat and often occurs during/after intramedullary reaming and nailing in diaphyseal femur fractures. Among the mechanisms involved, the release of lipids associated with other causes than intramedullary direct releases following trauma has not been fully investigated.

Technically, the measurement of lipids on a large scale is based on analytical chemistry principles and technological tools, particularly mass spectrometry. Recently, analytical techniques have greatly advanced. They can be analysed both quantitatively and qualitatively, and multiple lipid classes and species have been described. These findings have led us to identify new lipid-based signalling molecules, reveal the underlying lipid-driven mechanisms responsible for pathophysiological conditions. Several potential lipid biomarkers for early diagnosis and prognosis of diseases have been reported, such as genetic disorders [20], obesity and diabetes [21], cardiovascular disease [22], cancer [23], Alzheimer’s disease [24, 25], and neurodegenerative disease [21, 26]. Tissue-specific lipid distribution revealed by these lipidomic techniques is also believed to contribute to analysis of the origin of lipid change after trauma [27]. These lipidomic techniques allow for assessing differences in the damaging mechanisms of cell walls, subcellular particles, and others that might stimulate inflammatory systems (neutrophils etc.,) and might, therefore, represent a missing link.

Previous studies indicate that lipids may contribute to the systemic physiological process during resuscitation and recovery after trauma [28, 29]. We made a hypothesis that changes in circulating lipids are associated with responsiveness to resuscitation, and lipids have a potential to be biomarkers for detecting occult hypoperfusion. To our knowledge, only a few research studies have been reported investigating lipidomic changes after trauma and responsiveness to resuscitation. The current study is the first to do so in a controlled setting using a standardized porcine polytrauma model. The purpose of this study is to investigate the relation between the responsiveness to resuscitation and lipidomic course after trauma.

## Methods

### Trauma model

This study was conducted as a part of a research project using traumatized porcine models and data set which was analysed in this manuscript have not been included in any published literature [30]. The experimental model is based on previous porcine trauma models from our group [31]. Briefly, experiments were performed on 25 Swiss male large white pigs (Swiss landrace) weighing 50 ± 5 kg. Animals were exposed to standardized unilateral femoral fracture, blunt chest trauma with a lung contusion, grade II (American Association for the Surgery of Trauma) liver laceration, and pressure controlled haemorrhagic shock. Prior to the start of the experiment, all animals received premedication with an intramuscular injection of ketamine (Ketasol®—100, Dr. E. Graeub AG, Berne, Switzerland) 15 mg/kg, midazolam (Dormicum®, Roche Pharma (Schweiz) AG, Reinach, Switzerland) 0.5 mg/kg and atropine 0.05 mg/kg. Then, general anaesthesia was induced and maintained with a mixture of propofol (Propofol-® Lipuro, B. Braun Medical AG, Sempach, Switzerland; 5–10 mg/kg/h, constant rate infusion) and sufentanil forte (Sufenta® Forte, Janssen-Cilag AG, Zug, Switzerland; 0.01 mg/kg/h, constant rate infusion). Percutaneous placement of a femoral arterial line and a femoral two-lumen central venous catheter (HighFlow Dolphin Catheter, 13F, Baxter International, Deerfield, Illinois, United States) were performed. Crystalloids (Ringerfundin 2 ml/kg BW/h) were administered continuously and haemodynamics and metabolics were checked at set time points. A bolt gun machine (Blitz-Kerner, turbocut JOBB GmbH, Germany) and a custom-made metal plate were utilized to produce a standardized left-sided midshaft transverse femur fracture under sterile conditions. Fracture induction was confirmed by fluoroscopy. Liver laceration was performed using a custom-made star-shaped knife for stabbing the left lobe. The injury was treated with liver packing. Moderate blunt chest trauma was performed using a bolt gun and a lead plate for injury distribution. Mean atrial pressure (MAP)-controlled haemorrhagic shock was introduced (30 ± 5 mm Hg for 60 min). One hour after trauma, animals were resuscitated according to established trauma guidelines (ATLS, AWMF‐S3 Guidelines on Treatment of Patients with Severe and Multiple Injuries) by adjusting fraction of inspiratory oxygen and infusing additional fluids (Ringerfundin, 2 ml/kg body weight/h) [32, 33]. All animals were rewarmed until normothermia (38.7–39.8 °C) was reached. Fracture stabilization was also performed with a standardized nailing system and a shortened (80 mm) human distal femoral nail (cannulated DFN Ø 8.0 mm, DePuySynthes, Raynham, Massachusetts, United States).

### Vital parameter monitoring, lactate, and lipidomic analysis

Basic vital parameters, including heart rate (HR), systolic blood pressure (SBP), MAP, and oxygen saturation (SaO2), were recorded, and a venous blood sample for lactate and lipidomic analysis was collected at six time points: baseline (BL), during trauma and haemorrhagic shock (Trauma), 1 h after trauma (1 h), 2 h after trauma (2 h), 4 h after trauma (4 h), and 6 h after trauma (6 h). Furthermore, the shock index (SI) was obtained by dividing HR by SBP at each time point. Serum and plasma were separated by centrifugation immediately after the blood draw and stored frozen. Lipid extraction and liquid chromatography coupled mass spectrometry was carried out as described [34]. Lipids were separated using a C30 Accucore LC column (150 mm × 2.1 mm, 2.6 µm particle size) and a Transcend UHPLC pump (Thermo Fisher Scientific). Mass spectrometry analysis was done on a hybrid quadrupole–orbitrap mass spectrometer (Q-Exactive, Thermo Fisher Scientific). Lipid identification criteria were: (i) resolution with an accuracy of 5 ppm from the predicted mass at a resolving power of 70,000 at 200 m/z, (ii) isotopic distribution, (iii) expected retention time, and (iv) fragmentation pattern. Data analysis was performed using Tracefinder 5.1 (Thermo Fisher Scientific) for peak picking, annotation and matching to an in-house lipid database. Lipids were stratified into 15 subgroups based on molecular characteristics as follows: acylcarnitines (AcCas), cholesteric esters (CEs), ceramides (Cers), glucosylceramides (CerGs), coenzyme Q10s, diacylglycerols (DAGs), fatty acids (FAs), lysophosphatidylcholines (LPCs), phosphatidylcholines (PCs), phosphatidylethanolamines (PEs), phosphatidylglycerols (PGs), phosphatidylinositols (PIs), phosphatidylserines (PSs), sphingomyelins (SMs), and triglycerides (TGs). Blood dilution during the experiment was normalized for albumin [35].

### Responder group and occult hypoperfusion group

Animals were divided into two groups based on serum lactate level at the end point of observation (6 h) as an indicator of responsiveness to resuscitation. We chose a serum lactate level of 2 mmol/L as the threshold for the presence of occult hypoperfusion according to the previous study reported by Caputo et al. [9]. Animals with a serum lactate level <2 mmol/L at 6 h were defined as the responder group (R group), and animals with a serum lactate level ≥2 mmol/L at 6 h were defined as the occult hypoperfusion group (OH group).

### Statistical analysis

Data are presented as mean ± standard error (SE). Python programming language version 3.7.12 (https://www.python.org/) and SPSS version 26 (IBM, Chicago, Illinois, United States) were used for statistical analysis. To assess the definition of the occult hypoperfusion we employed in this study, we performed comparison of mean vital parameters and serum lactate level on each time point between the R group and OH group with unpaired *t* test. To detect the difference in patterns of lipidomic changes between two groups, mean lipid concentrations were compared between BL as a control and other time points in each group with Dunnett’s test. A *p* value <0.05 was considered statistically significant. Post hoc power analysis was performed with G*power software Version 3.1.9.6 (http://www.gpower.hhu.de/) and the Bonferroni controlling procedure was employed as necessary.

## Results

**Fig. 1:**
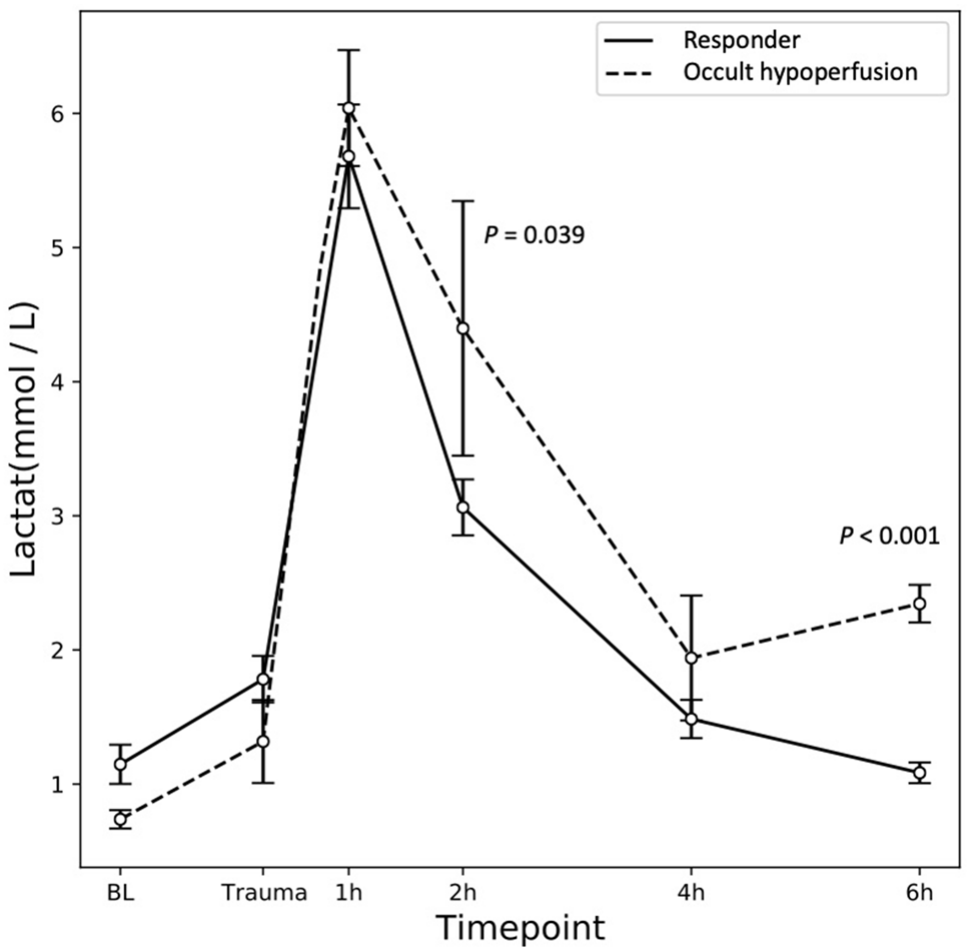
Serum lactate levels show a peak at 1 h in both groups. The occult hypoperfusion group is significantly greater than the responder group at 2 h after trauma (2 h) (*p* = 0.039) and at 6 h after trauma (6 h) (*p* < 0.001)

Among total of 25 animals, 18 animals (72%) met criteria for the R group, 4 animals (16%) for the OH group, and 3 animals (12%) died during the observation period. Vital parameters did not show any significant differences between the R group and OH group at any time point; HR (Trauma: 98.22 ± 4.05 vs. 86.75 ± 10.21, *p* = 0.255; 6 h: 99.61 ± 5.60 vs 102.75 ± 9.10, *p* = 0.808), SBP (Trauma: 38.39 ± 3.06 mmHg vs 46.75 ± 6.77 mmHg, *p* = 0.261; 6 h: 63.33 ± 2.84 mmHg vs 64.25 ± 5.07 mmHg, *p* = 0.889), MAP (Trauma: 30.56 ± 1.93 mmHg vs 37.25 ± 4.77 mmHg, *p* = 0.165; 6 h: 47.39 ± 1.65 mmHg vs 47.50 ± 3.38 mmHg, *p* = 0.977), SaO_2_ (Trauma: 97.44 ± 0.83% vs 98.75 ± 0.48%, *p* = 0.480; 6 h: 98.89 ± 0.36% vs 98.75 ± 0.75%, *p* = 0.871), SI (Trauma: 2.82 ± 0.15 vs 2.08 ± 0.10, *p* = 0.182; 6 h: 1.65 ± 0.14 vs 1.61 ± 0.14, *p* = 0.917). Serum lactate levels showed a peak at 1 h in both groups. Lactate levels were significantly higher in the OH group than in the R group at the 2 h and 6 h time points (2 h: 3.06 ± 0.21 mmol/l vs 4.40 ± 0.72 mmol/l, *p* = 0.039; 6 h: 1.08 ± 0.08 mmol/l vs 2.35 ± 0.11 mmol/l, *p* < 0.001) (Fig. 1).

**Fig. 2:**
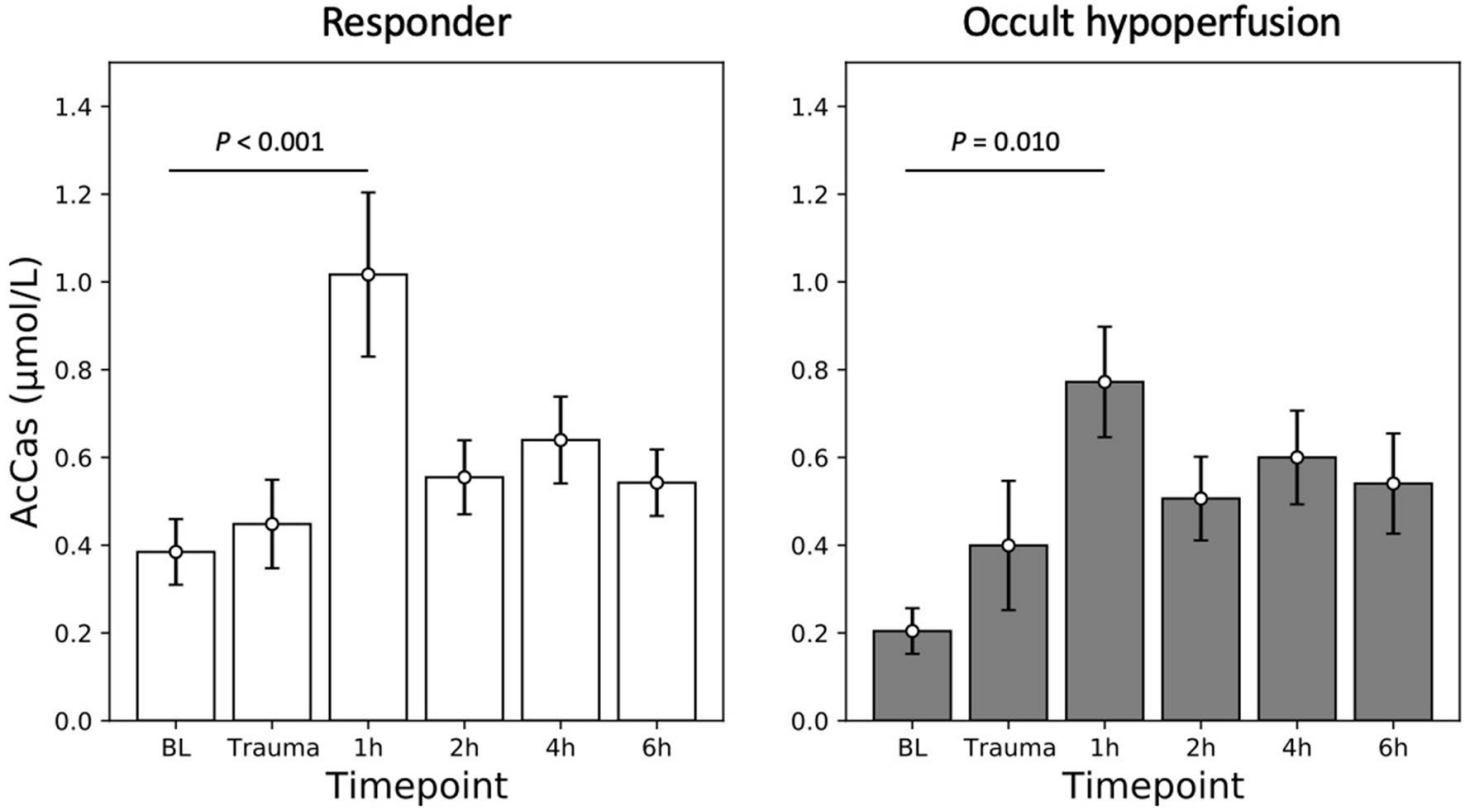
Acylcarnitines (AcCas) concentration shows a significant increase at 1 h after trauma (1 h) compared with that at base line (BL) in both groups (*p* < 0.001 and *p* = 0.010)

**Fig. 3:**
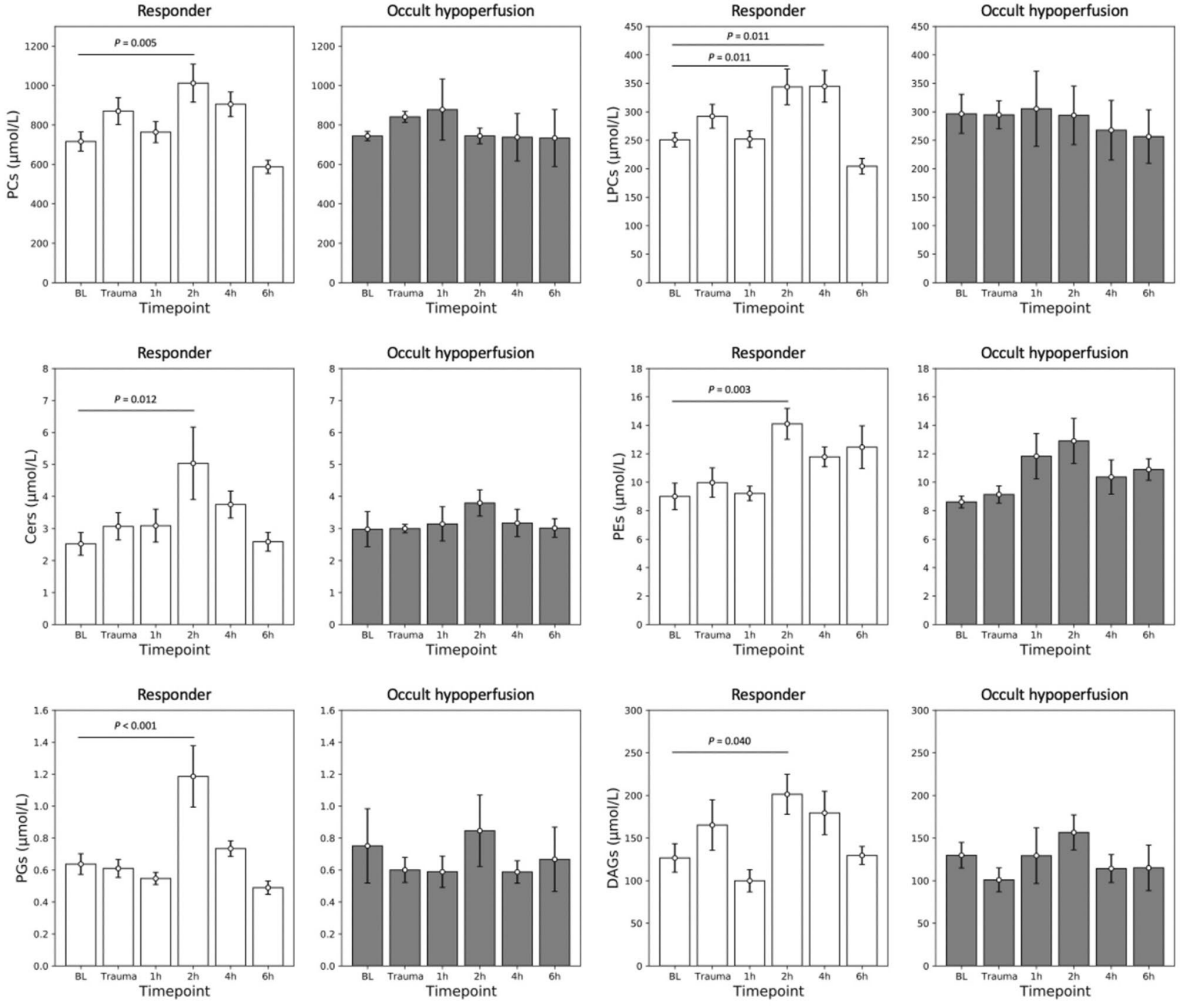
Concentration of six lipid groups shows a significant increase in the responder group at 2 h after trauma (2 h) compared with that at base line (BL) (phosphatidylcholines (PCs): *p* = 0.005, lysophosphatidylcholines (LPCs): *p* = 0.011, ceramides (Cers): *p* = 0.012, phosphatidylethanolamines (PEs): *p* = 0.003, phosphatidylglycerols (PGs): *p* < 0.001, and diacylglycerols (DAGs): *p* = 0.040), and no significant change of these lipids was observed in the occult hypoperfusion group

Lipids have shown different patterns of response after severe trauma. AcCas showed a significant increase at 1 h compared with that at BL in both groups (R group: 0.38 ± 0.07 μmol/l vs 1.01 ± 0.19 μmol/l, *p* < 0.001; OH group: 0.20 ± 0.05 μmol/l vs 0.77 ± 0.13 μmol/l, *p* = 0.010) (Fig. 2). Other lipid groups, however, have shown a different pattern of response, including PCs, LPCs, Cers, PEs, PGs, and DAGs, demonstrating significant elevations in the R group at 2 h compared with that at BL (PCs: 716.27 ± 48.78 μmol/l vs 1012.32 ± 96.50 μmol/l, *p* = 0.005; LPCs: 250.88 ± 12.55 μmol/l vs 343.79 ± 31.30 μmol/l, *p* = 0.011; Cers: 2.52 ± 0.36 μmol/l vs 5.03 ± 1.13 μmol/l, *p* = 0.012; PEs: 9.00 ± 0.93 μmol/l vs 14.11 ± 1.09 μmol/l, *p* = 0.003; PGs: 0.64 ± 0.06 μmol/l vs 1.19 ± 0.19 μmol/l, *p* < 0.001; DAGs: 126.65 ± 16.72 μmol/l vs 201.41 ± 23.53 μmol/l, *p* = 0.040). There were no significant changes of lipids in the OH group during the whole course of observation (Fig. 3).

## Discussion

Severe trauma accounts for 8% of deaths worldwide [36]. Among the surviving patients submitted to resuscitation, occult hypoperfusion has been discussed to occur in 68% [37]. In this line, our study demonstrated the following results:

- Occult hypoperfusion occurred in a substantial number of animals (16%) despite adequate volume replacement, as determined in our endpoints of resuscitation.
- The occurrence of occult hypoperfusion seemed to be related to lipid release patterns, as significant increases of AcCas concentrations were observed in both groups at 1 h, whereas six lipid groups (PCs, LPCs, Cers, PEs, PGs, and DAGs) were increased at 2 h only in the R group.

It has repeatedly been observed that a persistently high lactate level, despite aggressive resuscitation, is a strong predictor of multiple organ failure and mortality [38]. Therefore, clinical use of the serum lactate level as a diagnostic tool to assess the patient’s status and make treatment strategy decisions have been reported in various studies [39]. In the current study, there was no difference in vital parameters at the end of resuscitation between two groups. Nevertheless, even after a standardized resuscitation in a standardized trauma model, a high number of animals showed high lactate level, which indicated that they did not have sufficient perfusion.

In the current study, PCs, LPCs, Cers, PEs, PGs, and DAGs increased at 2 h only in group R. These results indicate that there is possibility of relation between perfusion and lipid release. Although most lipid functions are still unclear, two possible explanations for these lipidomic courses can be considered. First, the R group could show a physiological reaction to insults, which contribute to successful recovery, including generation or release of lipids. DAGs and PEs are linked though a common pathway, which may explain why their levels increased at the same time [40]. DAGs possibly act as a supplier not only of energy but also of precursors for lipid mediators involved in inflammation resolution and tissue repair [41–43]. This kind of physiological reaction could be insufficient because of organ failure after severe hypoperfusion damage in the OH group [44]. The other possible explanation could be, that a lipid leakage from damaged organs and tissues into the circulation, as known from the pathophysiology of fat embolism syndrome [45]. These lipids are highly concentrated in the following organs and tissues in the body: DAGs in subcutaneous adipose tissue, visceral adipose tissue, liver, and kidney; PCs, LPCs, PEs and Cers in liver, kidney, and heart; and PGs in liver and kidney [27]. Loss of haemodynamic coherence between the macro- and microcirculation after severe hypoperfusion damage could delay the lipid transportation from these organs and tissues to circulation in the OH group [46]. In contrast to these six lipids, AcCas was increased in both groups immediately after trauma (at 1 h) in the current study. One may wonder whether AcCas could represent a potential biomarker, as AcCas represents a lipid subgroup formed in mitochondria. These are generally considered to be a transport form of FAs and can be utilized for energy production in mitochondria or for the synthesis of endogenous molecules [47, 48]. Previous studies have shown increased plasma AcCas in patients with congenital FA oxidation disorders, patients with sepsis, and rat controlled cortical impact models [49–51]. We suspect that mitochondrial dysfunction in peripheral tissue cells and subsequent transportation of AcCas to the circulation caused by hypoxic damage in the hypoperfusion condition during haemorrhage shock could be one of the possible explanations for increased concentrations of this lipid after trauma in this study.

This study had some limitations. First, results in this study cannot be applied to clinical cases directly because our poly trauma model demonstrates only one of the patterns of wide varieties of trauma. On the other hand, our model, which was designed and established to represent multiple trauma condition with suspected hypoperfusion, involves haemorrhagic shock, which is the most major and direct cause of hypoperfusion in traumatized patients, and also femoral fracture, lung contusion, and liver laceration, which are most common injuries and can influence the systemic condition [31]. Therefore, we believe that the results observed from this model can contribute to the research on systemic reaction to trauma and resuscitation. Unspecific character of serum lactate level seemed to be one of limitations as well. However, we decided to employ this parameter for grouping because multiple previous studies have described elevated lactate level with stabilized macrocirculation as the definition of occult hypoperfusion [9, 11–15]. In addition, animals were divided into two groups after trauma and resuscitation, and, therefore, resulting imbalanced number of animals between groups. Although these might be other limitations, considering the nature of occult hypoperfusion, grouping after trauma and resuscitation is the only way to make occult hypoperfusion animal model and the risk of imbalanced number of animals is unavoidable. Furthermore, measuring other parameters which may have an impact on the lactate level or the circulating lipids, such as lactate dehydrogenase and catecholamines in circulation, might have given us more knowledge to clarify the pathology of occult hypoperfusion. Another limitation is that animals were observed only until the 6 h after trauma. Further physiological changes are known to occur after this end point [28]. Additionally, further investigations were desirable to elucidate the mechanisms of changes in circulating lipids after trauma and resuscitation. Our speculation that increases of circulating AcCas in both groups might be a result of mitochondrial dysfunction could be supported by analysing mitochondrial function, or mitochondrial DNA in the circulation. Moreover, employing additional animal group without nailing to the measurements in the current study could give us the information to estimate the influence from bone marrow manipulation to circulating lipids composition.

In conclusion, we investigated the relation between responsiveness to resuscitation and lipidomic course after trauma in a standardized polytrauma porcine model. Occult hypoperfusion occurred in 16% in this highly standardized model. Six lipid groups (PCs, LPCs, Cers, PEs, PGs, and DAGs) increased significantly only in the R group at 2 h, which may support the idea that they could serve as potential biomarkers. We feel that further study is required to confirm the role of lipid release in the systemic physiological changes after trauma.

## Data Availability

The original dataset generated during the current study is available from the corresponding author on reasonable request.
